# Smart Nanovesicles for Drug Targeting and Delivery

**DOI:** 10.3390/pharmaceutics11040147

**Published:** 2019-03-29

**Authors:** Carlotta Marianecci, Maria Carafa

**Affiliations:** Dipartimento di Chimica e Tecnologie del Farmaco, Sapienza University of Rome, 00185 Rome, Italy

Nanovesicles are highly-promising and versatile systems for the delivery and/or targeting of drugs, biomolecules and contrast agents. Despite the fact that initial studies in this area were performed on phospholipid vesicles, there is an ever-increasing interest in the use of other molecules to obtain smart vesicular carriers focusing on strategies for targeted delivery. This special issue aims to highlight and capture the contemporary progress and current landscape of smart nanovesicles applied in drug targeting and delivery.

A series of research articles and one review are present in this special issue and offer a summary of the different researches by different countries’ teams, thus making meaningful and significant contributions to the field.

Asprea et al. investigated the possibility to obtain monodisperse and stable nanocochleates from Natural Soy Lecithin Liposomes, using two different phospholipids, phosphatidylcholine and phosphatidylserine, loaded with a typical small hydrophobic natural product, andrographolide (AG). AG from the Asiatic medicinal plant *Andrographis paniculata* shows numerous potential activities ranging from anti-inflammatory to neuroprotection, antidiabetic to anti-obesity properties, and antitumor activity to hepatoprotective activity. It has poor water solubility which deeply limits its biodistribution and localization, resulting in low bioavailability and additionally, is unstable in gastrointestinal media and has a very short biological half-life (*t*_½_ = 1.33 h) after a single oral dose. The stability of developed nanocochleates after lyophilisation and in simulated gastrointestinal fluids was investigated. In addition, the studied nanocarriers show high EE%, and suitable drug release properties for oral delivery, but with possible uses in other routes of administration [1].

In a second study Piazzini et al. evaluated the possibility of using liposomes to enhance the penetration into the brain of AG. The AG-loaded liposomes showed protection against damage induced by amyloid-oligomers in vitro, reduction of amyloid levels and tau phosphorylation in mice, modulation of the formation of amyloid plaques and recovery of spatial memory functions in Alzheimer’s disease transgenic mouse model. Liposomal surface was modified by adding Tween 80 alone or in combination with Didecyldimethylammonium bromide to confer cationic surface charge. Liposomes were evaluated for various formulation parameters (size, polydispersity, ζ-potential, morphology, chemical and physical stability, in vitro release) and the optimized formulations were studied and characterized with in vitro tests. Both formulations enhanced solubility and cellular permeability of AG, as in vitro tests with PAMPA and hCMEC/D3 cells and increase the permeation of AG into the cell without alterations in cell viability and monolayer integrity. The presence of positive charge elevated the cellular internalization of liposomes [2].

Another interesting study on a natural compound is the one by Santos-Rebelo and colleagues. In this research study, Parvifloron D was efficiently extracted and isolated from *P. ecklonii* and it showed more selectivity to human pancreatic tumor cells than healthy cells or breast cancer cells, but Parvifloron D is affected by low water-solubility, thus, small and spherical albumin nanoparticles (water soluble particles) have been formulated with high encapsulation efficiency to enhance drug solubility and targeted delivery. Those nanoparticles led to a controlled release of the drug, which was stable, and therefore, they can be considered a suitable and promising carrier to deliver the drug to the tumor site, improving the treatment of pancreatic cancer [3].

The great interest around natural compound delivery was confirmed by the study reported by Di Sotto and colleagues. They performed a deep physical-chemical characterization of soybean phosphatidylcholine (SPC) liposomes used to improve the dissolution of the natural sesquiterpene-caryophyllene (CRY) in biological fluids and its cellular uptake. Both unilamellar (ULV) and multilamellar (MLV) formulations were studied. The lipid composition, lamellarity, the manufacturing process and drug incorporation can all influence the physicochemical properties of a liposomal formulation, including the drug release performance. In particular, the influence of the drug–lipid ratio on the arrangement of the nonpolar region of the vesicles’ membrane must be considered to design a carrier able to entrap and then release the loaded drug to obtain the therapeutic effect. The antiproliferative activity of CRY-loaded SPC ULV and MLV with respect to that of CRY alone was also studied in liver cancer HepG2 cells and MDA-MB-468 [4].

In the research study carried out by Coccè and colleagues, the application of extracellular vesicles in the paclitaxel delivery was evaluated. In particular, the anticancer activity of secretomes from both untreated and paclitaxel (PTX)-primed GinPaMSCs, by demonstrating that both PTX-loaded GinPaMSCs and the corresponding extracellular vesicles (EVs/PTX) were active against cancer cells. This research study provides a strong proof of concept, suggesting a possible application of the procedure to collect PTX-associated EVs from drug-primed GinPaMSC working as “natural anticancer liposomes” [5].

The study of Palchetti and colleagues focused on an important aspect related to liposomal administration: the understanding that the limited success of liposomal drugs in clinical practice is due to our poor knowledge of the nano–bio interactions experienced by liposomes in vivo. In this study, a library of 10 liposomal formulations with systematic changes in lipid composition were prepared and exposed to human plasma. Size, zeta-potential, and corona composition of the resulting liposome–protein complexes were thoroughly characterized. According to the recent literature, enrichment in protein corona fingerprints (PCFs) was used to predict the targeting ability of synthesized liposomal formulations. In this study, the predicted targeting capability of liposome–protein complexes was clearly correlated with cellular uptake in pancreatic adenocarcinoma (PANC-1) and insulinoma (INS-1) cells. The cellular uptake of the liposomal formulation with the highest abundance of PCFs was found to be much larger than that of Onivyde^®^, an Irinotecan liposomal drug approved by the Food and Drug Administration in 2015 for the treatment of metastatic pancreatic ductal adenocarcinoma [6].

An example of a pH sensitive targeting by using non-ionic surfactant vesicles is represented by the research study by Marzoli and colleagues. The anti-inflammatory and analgesic activity in acute and chronic models of pain of ibuprofen loaded pH sensitive vesicles was evaluated. These niosomes, with increased affinity for an acidic pH microenvironment, can take advantage of pathological conditions (ischemia, infection, inflammation, and cancer where extracellular pH values range from 5.5 to 7.0) for selective targeting. In particular pH-Tw20Gly niosomes loaded with ibuprofen were compared to free ibuprofen in animal models of acute and chronic pain. pH sensitive niosomal formulations increase Ibuprofen’s analgesic activity, promoting a longer duration of action of this drug [7].

In the study of Rodrigues et al., multifunctional liposomes containing manganese ferrite/gold core/shell nanoparticles were developed in order to obtain simultaneous chemotherapy and phototherapy. In order to develop applications in cancer therapy, the prepared nanoparticles were entrapped in liposomes (aqueous magnetoliposomes, AMLs) or covered with a lipid bilayer (solid magnetoliposomes, SMLs). These new nanosystems were tested in this scenario as nanocarriers for a potential anticancer drug, especially active against melanoma, breast adenocarcinoma, and non-small cell lung cancer. The local heating capability of the developed systems was also monitored [8].

An alternative route of administration by means of a nanotechnological strategy was proposed by Touitou and colleagues for buspirone delivery. In particular, the nasal administration of buspirone incorporated in a new nanovesicular delivery system (NDS) to be tested in a hot flushes animal model was studied. The role of the carrier in the design of an efficient nasal product is fundamental, so to this aim, in this work, buspirone NDS was appropriately designed and extensively characterized, then the pharmacodynamic effect in an ovariectomized (OVX) animal model for hot flushes, and the drug levels in brain and plasma were evaluated. The safety of the local application of the nanovesicular system on the animal nasal cavity was also examined [9].

Finally, the review by Narayan and colleagues reported an overview on mesoporous silica nanoparticles (MSNs), a material with high thermal, chemical and mechanical properties, that have garnered immense attention as drug carriers owing to their distinctive features over the others [10].

All the articles presented in the special issue represent a small cross-section of a great research interest in the field of nanovesicular system applications in drug delivery.

From the overall presented results, several interesting potentialities of these systems have been highlighted together with their high versatility and excellent biocompatibility. These qualities make them attractive and we hope that they will soon be able to represent an evolution in products available on the market.
